# Identifying High-Risk Psychiatric Subgroups for Readmission Among Heart Failure Patients: A Seven-Year National Analysis in the United States

**DOI:** 10.7759/cureus.88243

**Published:** 2025-07-18

**Authors:** Kyle E Thurmann, Trisha G Mukherjee, Joseph G Dantin, Paul Kang, Michael D White

**Affiliations:** 1 School of Medicine, Creighton University School of Medicine, Phoenix, USA; 2 School of Medicine, Rocky Vista University College of Osteopathic Medicine, Parker, USA; 3 Cardiology, Creighton University School of Medicine, Omaha, USA

**Keywords:** bipolar disorder, heart failure, hospital readmission, psychiatric comorbidities, ptsd, schizophrenia, substance use disorder, transitional care

## Abstract

Background

Psychiatric comorbidities are common among patients with heart failure (HF) and may significantly influence hospital readmissions. While prior studies have linked mental illness to readmission risk, few have clarified which specific psychiatric diagnoses confer the greatest risk, limiting the development of diagnosis-specific interventions. Leveraging the nationally representative Nationwide Readmissions Database (NRD), this study aimed to define these associations and inform diagnosis-specific interventions.

Methods

We conducted a retrospective cohort study using the 2016-2022 NRD. Adults hospitalized for HF were identified using ICD-10-CM codes. Patients who died, left against medical advice, were transferred, had planned readmissions, or had missing critical data were excluded. Psychiatric comorbidities were defined using ICD-10 codes (primary and secondary diagnoses) for depression, anxiety disorders, bipolar disorder, schizophrenia/psychotic disorders, post-traumatic stress disorder (PTSD), and substance use disorder (SUD). The primary outcome was unplanned all-cause readmission at 30 days and one year. We used multivariable Cox proportional hazards models adjusted for age, sex, insurance type, income quartile, hospital characteristics, discharge disposition, and the Charlson Comorbidity Index. Adjusted HRs and 95% CIs were reported.

Results

Among 31,886,859 weighted HF hospitalizations, schizophrenia or psychotic disorders (HR: 1.05; 95% CI: 1.05-1.06), bipolar disorder (HR: 1.03; 95% CI: 1.02-1.04), PTSD (HR: 1.03; 95% CI: 1.02-1.04), and SUD (HR: 1.02; 95% CI: 1.02-1.03) were each independently associated with increased 30-day all-cause readmission (p < 0.001 for all), while depression (HR: 0.99; 95% CI: 0.98-0.99; p < 0.001) was associated with reduced risk, and anxiety (HR: 0.99; 95% CI: 0.97-1.02; p = 0.86) showed no significant association. At one year, schizophrenia or psychotic disorders (HR: 1.09; 95% CI: 1.08-1.10), bipolar disorder (HR: 1.06; 95% CI: 1.05-1.07), PTSD (HR: 1.05; 95% CI: 1.04-1.06), and SUD (HR: 1.04; 95% CI: 1.03-1.04) remained significantly associated with increased risk (p < 0.001 for all), whereas depression (HR: 0.99; 95% CI: 0.99-1.01; p = 0.69) and anxiety (HR: 1.00; 95% CI: 0.98-1.01; p = 0.99) were not.

Conclusions

Schizophrenia, bipolar disorder, PTSD, and SUD were independently associated with elevated short- and long-term all-cause readmission following HF hospitalization. These findings underscore the importance of incorporating psychiatric risk factors into diagnosis-specific transitional care strategies for adults hospitalized with HF.

## Introduction

Psychiatric illness is increasingly recognized as a significant contributor to worse cardiovascular (CV) outcomes. Among young adults aged 18 to 25, the prevalence of any mental illness (e.g., mild depression or anxiety) increased from 18.5% (6.1 million) in 2008 to 29.4% (9.9 million) in 2019, while serious mental illness (e.g., schizophrenia or major depression with severe impairment) more than doubled from 3.8% to 8.6% (2.9 million) [1]. During the COVID-19 pandemic, the percentage of US adults reporting symptoms of anxiety and depression surged from 11% in 2019 to between 31.5% and 39.3% by early 2023, alongside a sharp rise in substance use and overdose deaths, with over 100,000 overdose fatalities reported in 2022 [2]. Together, these trends highlight an expanding burden of psychiatric illness across both younger and older adult populations in the US.

These rising trends in mental illness, particularly in younger populations, underscore the importance of understanding their direct links to CV outcomes. Large-scale analyses have consistently demonstrated that mental health conditions, including major depression, bipolar disorder, anxiety disorders, post-traumatic stress disorder (PTSD), and schizophrenia, are independently associated with adverse CV outcomes. For example, a meta-analysis of over three million individuals reported markedly higher rates of cardiovascular disease (CVD) incidence and mortality in patients with schizophrenia, bipolar disorder, or major depression [3]. Additionally, a nationwide analysis found that psychiatric disorders conferred an elevated risk of readmission after acute myocardial infarction, with schizophrenia presenting the highest risk [4]. Substance use disorders (SUDs) further compound CV risk, particularly in those with opioid, stimulant, or alcohol-related conditions, with CVD-related mortality in this population more than doubling between 1999 and 2019 [5-7]. Although these six psychiatric comorbidities have been linked to adverse CV outcomes, few studies have evaluated their specific association with heart failure (HF) readmission, particularly across multiple timepoints in a large, nationally representative adult cohort.

HF remains one of the most frequent and costly causes of hospitalization in the US, with projected healthcare expenditures nearing $70 billion by 2030. Readmissions account for a substantial portion of this economic burden [8-10]. Coinciding with the rise in mental illness among younger adults, HF-related mortality is also increasing in this group, driven by emerging risk factors such as metabolic syndrome, environmental exposures, and substance use [11]. Hospitalizations for HF among adults aged 18-44 years in the US increased by 23% between 1999 and 2019, with 1%-3% of all HF cases now occurring in individuals under 40 years, with increased rates among young Black adults [11,12]. These developments underscore the urgent need to identify modifiable contributors to early and recurrent HF hospitalizations, particularly in vulnerable populations. While younger adults represent a growing share of HF and psychiatric illness burden, our study includes all adults aged 18 and older to provide generalizable findings across the adult HF population and to provide a foundation for future age-stratified research.

To address the limited understanding of how distinct psychiatric comorbidities influence HF readmissions, we conducted one of the largest nationally representative analyses to date using the Nationwide Readmissions Database (NRD). This study aimed to identify which of six psychiatric conditions (depression, anxiety disorders, bipolar disorder, schizophrenia or other psychotic disorders, PTSD, and SUD) are most strongly associated with short-term (30-day) and long-term (one-year) all-cause readmissions following HF hospitalization among adults aged 18 and older. We hypothesized that severe mental illnesses such as schizophrenia, bipolar disorder, PTSD, and SUD would be independently associated with higher readmission risk, based on prior evidence linking these conditions to adverse CV outcomes. These diagnoses were selected based on their high national prevalence, known CV risks, and clinical relevance for integrated psychiatric and CV care [3-7]. While the primary aim was to assess diagnosis-specific readmission risk, we also characterized sociodemographic and healthcare system factors within the overall HF population to contextualize patterns of psychiatric comorbidity and inform targeted intervention strategies.

## Materials and methods

Data source

This study utilized data from January 2016 to December 2022 using the NRD, a component of the Healthcare Cost and Utilization Project (HCUP) supported by the Agency for Healthcare Research and Quality. The NRD compiles hospital discharge records from 30 diverse states, covering about 61% of the US population and 60% of hospitalizations nationwide [13]. Each patient is assigned a unique, synthetic linkage variable that allows tracking of hospitalizations within a given calendar year, but not across years.

Study population

We included individuals aged 18 years and older who were admitted with a principal diagnosis of HF, identified using ICD-10-CM codes I50.1 through I50.9. We excluded patients who (1) died during the index hospitalization; (2) were transferred to another acute care hospital; (3) left against medical advice; (4) had planned readmissions; or (5) had missing critical demographic or outcome variables. Less than 1% of otherwise eligible discharges were excluded due to missing data; imputation was not performed. To ensure independence of index events, only the first eligible HF hospitalization per patient per calendar year was included. All exclusion criteria are detailed in-text to maintain clarity without requiring a flow diagram. While the same NRD cohort was used in two other independent investigations, this study addresses a distinct research question with non-overlapping exposures, outcomes, and analytic aims [14,15].

Definition of psychiatric comorbidity

To assess the independent impact of psychiatric comorbidities, we identified patients with at least one psychiatric condition using ICD-10 codes for depression (F32.0-F32.9, F33.0-F33.9), anxiety disorders (F40.0-F41.9), bipolar disorder (F31.0-F31.9), schizophrenia or other psychotic disorders (F20.0-F20.9, F25.0-F25.9), PTSD (F43.10-F43.12), and SUDs (F10-F19, including all subtypes). Psychiatric diagnoses were based solely on codes present during the index HF admission; historical diagnoses from prior encounters were not available and therefore not included. Diagnostic groups were not mutually exclusive. Patients with multiple psychiatric diagnoses were included in all applicable categories, and each diagnosis was modeled as a separate binary variable in the multivariable models. This approach allowed for the independent assessment of each condition’s association with readmission risk while accounting for potential diagnostic overlap.

Outcome measures

The primary endpoint was all-cause hospital readmission within 30 days and one year after discharge from the index HF hospitalization. Only unplanned readmissions were included. Planned readmissions were excluded using HCUP’s standard readmission classification variable. Readmission events were determined using unique linkage variables in the NRD that track de-identified patients across admissions within a calendar year.

Covariates

We extracted patient demographics (age, sex, ZIP code-based income quartile, and primary payer), hospital-level features (urban vs. rural location, teaching status, and bed capacity), and clinical characteristics. Admission timing (weekend vs. weekday) and discharge disposition (home, skilled care, home health, or institutional facility) were recorded. The Charlson Comorbidity Index (CCI), as defined in the NRD, was used to quantify comorbidity burden. This validated tool is commonly employed to estimate mortality risk and healthcare utilization in administrative datasets [16].

Statistical analysis

All analyses incorporated HCUP-provided survey weights to generate nationally representative estimates and accounted for the complex sampling design of the NRD, including clustering and stratification, using appropriate variance estimation procedures. We used weighted chi-squared tests to compare categorical variables. Cox proportional hazards models were constructed to evaluate associations between psychiatric conditions and all-cause readmissions at both follow-up intervals. Adjusted HRs and 95% CIs were reported, controlling for demographic, hospital, and clinical covariates, including CCI. Proportional hazards assumptions were verified using Schoenfeld residuals and found to be met. To account for hierarchical data structure, we performed sensitivity analyses using robust SEs clustered at the hospital level; results were materially unchanged. Records with missing key variables were excluded; less than 1% of eligible discharges were removed due to missing data. A p-value threshold of <0.001 defined statistical significance.

Software and ethics compliance

Analyses were performed using STATA version 18 (StataCorp LLC, College Station, TX, USA). All procedures adhered to HCUP and NRD data use policies.

## Results

Our analysis included 31,886,859 weighted hospitalizations for HF from the NRD. The average patient age was 71.3 years (SE 0.004), and women comprised 48.9% (N = 15,592,674) of the cohort. The majority of patients were covered by Medicare (75.7%, N = 24,138,352), lived in urban settings (82.4%, N = 26,274,772), and belonged to the lowest income quartile (32.8%, N = 10,458,890). More than half of the admissions occurred at large hospitals (53.8%, N = 17,155,130), with most patients treated at urban teaching institutions (69.8%, N = 22,257,028). Nearly 75% (N = 23,883,257) of patients had a CCI score of 3 or higher. At discharge, 45.6% (N = 14,540,408) returned home, 25.8% (N = 8,226,810) received home healthcare, and 25.7% (N = 8,194,923) were transferred to post-acute care facilities. The prevalence of psychiatric comorbidities in this population included depression (14.6%, N = 4,655,481), anxiety (0.29%, N = 92,472), bipolar disorder (2.1%, N = 669,624), schizophrenia or psychotic disorders (1.5%, N = 478,303), PTSD (0.8%, N = 255,095), and SUD (19.2%, N = 6,122,277). A detailed summary of these demographic and clinical characteristics is presented in Table 1.

**Table 1:** Baseline demographic, clinical, and psychiatric characteristics of the study population This table presents weighted baseline characteristics for 31,886,859 hospitalizations of adults with HF in the NRD. Notably, depression (14.6%, N = 4,655,481) and SUD (19.2%, N = 6,122,277) were the most common psychiatric diagnoses. CCI, Charlson Comorbidity Index; HF, heart failure; NRD, Nationwide Readmissions Database; PTSD, post-traumatic stress disorder; SUD, substance use disorder

Demographics (weighted)	Overall (N = 31,886,859)	
	% (N)	SE
Age, years	Mean = 71.3	0.004
Sex, female	48.9 (15,592,674)	0.013
Income quartile		
1	32.8 (10,458,890)	0.011
2	27.7 (8,832,660)	0.011
3	22.9 (7,302,091)	0.011
4	16.7 (5,325,105)	0.009
Primary payer		
Medicare	75.7 (24,138,352)	0.011
Medicaid	10.1 (3,220,573)	0.007
Private	10.0 (3,188,686)	0.008
Self-pay	1.79 (570,775)	0.003
Other	2.4 (765,285)	0.004
Weekend admission	24.4 (7,780,394)	0.011
Patient location		
Urban	82.4 (26,274,772)	0.008
Rural	17.6 (5,612,087)	0.008
Hospital location		
Urban/non-teaching	20.4 (6,504,919)	0.002
Urban/teaching	69.8 (22,257,028)	0.003
Rural	9.8 (3,124,912)	0.002
Hospital bed size		
Small	18.5 (5,899,069)	0.002
Medium	27.7 (8,832,660)	0.002
Large	53.8 (17,155,130)	0.003
CCI		
0-1	7.3 (2,327,741)	0.007
2	17.8 (5,675,861)	0.009
>3	74.9 (23,883,257)	0.011
Disposition at discharge		
Home	45.6 (14,540,408)	0.012
Short-term facility	1.2 (382,642)	0.003
Designated center	25.7 (8,194,923)	0.011
Home healthcare	25.8 (8,226,810)	0.011
Against medical advice	1.7 (542,077)	0.003
Unknown	0.047 (14,987)	6.5E-06
Psychiatric covariates		
Depression	14.6 (4,655,481)	0.009
Anxiety	0.29 (92,472)	0.001
Bipolar disorder	2.1 (669,624)	0.004
Schizophrenia/psychotic disorders	1.5 (478,303)	0.003
PTSD	0.8 (255,095)	0.002
SUD	19.2 (6,122,277)	0.01

Among psychiatric subgroups, schizophrenia or psychotic disorders (26.9%, N = 128,664) and bipolar disorder (26.1%, N = 174,772) had the highest all-cause 30-day readmission rates. Adjusted Cox regression showed that schizophrenia or psychotic disorders (HR: 1.05; 95% CI: 1.05-1.06; p < 0.001), bipolar disorder (HR: 1.03; 95% CI: 1.02-1.04; p < 0.001), PTSD (23.7%, N = 60,458; HR: 1.03; 95% CI: 1.02-1.04; p < 0.001), and SUD (22.7%, N = 1,389,757; HR: 1.02; 95% CI: 1.02-1.03; p < 0.001) were each independently associated with elevated 30-day all-cause readmission risk. In contrast, depression (21.6%, N = 1,005,584) was associated with a slight reduction in risk (HR: 0.99; 95% CI: 0.98-0.99; p < 0.001), while anxiety (21.1%, N = 19,512) showed no significant association (HR: 0.99; 95% CI: 0.97-1.02; p = 0.86).

**Figure 1:**
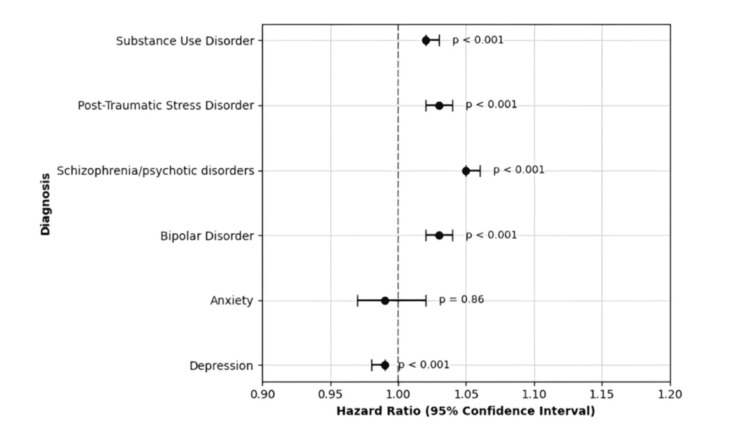
Adjusted HRs for 30-day all-cause readmission by psychiatric comorbidity This forest plot illustrates the multivariable-adjusted HRs and 95% CIs for 30-day all-cause readmission following HF hospitalization, stratified by psychiatric comorbidity. All HRs are derived from Cox proportional hazards models adjusted for patient demographics, socioeconomic status, and hospital characteristics. HF, heart failure

**Figure 2:**
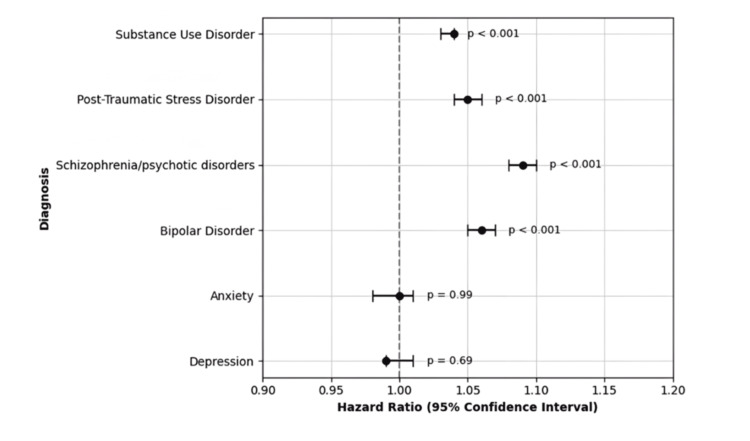
Adjusted HRs for one-year all-cause readmission by psychiatric comorbidity This forest plot displays the multivariable-adjusted HRs and 95% CIs for one-year all-cause readmission following HF hospitalization, stratified by psychiatric comorbidity. All HRs were derived from Cox proportional hazards models adjusted for patient demographics, socioeconomic status, and hospital characteristics. HF, heart failure

At one year, schizophrenia or psychotic disorders (51.7%, N = 247,283) and bipolar disorder (50.7%, N = 339,499) again had the highest all-cause readmission rates. These were followed by PTSD (46.8%, N = 119,384) and SUD (45.4%, N = 2,779,514). Each of these conditions remained significantly associated with increased one-year readmission: schizophrenia or psychotic disorders (HR: 1.09; 95% CI: 1.08-1.10), bipolar disorder (HR: 1.06; 95% CI: 1.05-1.07), PTSD (HR: 1.05; 95% CI: 1.04-1.06), and SUD (HR: 1.04; 95% CI: 1.03-1.04), all p < 0.001. Depression (44.6%, N = 2,076,345) and anxiety (43.1%, N = 39,855) were not significantly associated with increased one-year readmission risk (HR: 0.99, p = 0.69 and HR: 1.00, p = 0.99, respectively). The 30-day and one-year results are detailed in Table 2 and illustrated in Figure 1 and Figure 2.

**Table 2:** Risk of all-cause 30-day and one-year readmission by psychiatric comorbidity This table presents the weighted percentages and adjusted HRs for 30-day and one-year all-cause readmissions stratified by the presence or absence of psychiatric conditions from the NRD. Multivariable Cox regression was used to estimate HRs, adjusting for patient demographics, socioeconomic status, and hospital characteristics. Schizophrenia or psychotic disorders, bipolar disorder, post-traumatic stress disorder, and substance use disorder were associated with significantly increased hazards of all-cause readmission at both time points (p < 0.001). NRD, Nationwide Readmissions Database; PTSD, post-traumatic stress disorder; SUD, substance use disorder

Psychiatric condition	All-cause 30-day readmission				All-cause one-year readmission			
	% (N)	SE	HR (95% CI)	p-value	% (N)	SE	HR (95% CI)	p-value
Depression								
No	20.1 (935,752)	0.011	REF	<0.001	41.4 (1,927,369)	0.013	REF	0.69
Yes	21.6 (1,005,584)	0.028	0.99 (0.98, 0.99)		44.6 (2,076,345)	0.035	0.99 (0.99, 1.01)	
Anxiety								
No	20.3 (18,772)	0.01	REF	0.86	41.9 (38,746)	0.013	REF	0.99
Yes	21.1 (19,512)	0.2	0.99 (0.97, 1.02)		43.1 (39,855)	0.24	1.00 (0.98, 1.01)	
Bipolar disorder								
No	20.2 (135,264)	0.01	REF	<0.001	41.7 (279,233)	0.013	REF	<0.001
Yes	26.1 (174,772)	0.079	1.03 (1.02, 1.04)		50.7 (339,499)	0.091	1.06 (1.05, 1.07)	
Schizophrenia/psychotic disorders								
No	20.3 (97,096)	0.01	REF	<0.001	41.7 (199,452)	0.013	REF	<0.001
Yes	26.9 (128,664)	0.093	1.05 (1.05, 1.06)		51.7 (247,283)	0.11	1.09 (1.08, 1.10)	
PTSD								
No	20.3 (51,784)	0.01	REF	<0.001	41.8 (106,630)	0.013	REF	<0.001
Yes	23.7 (60,458)	0.12	1.03 (1.02, 1.04)		46.8 (119,384)	0.14	1.05 (1.04, 1.06)	
SUD								
No	19.8 (1,212,211)	0.011	REF	<0.001	41.1 (2,516,256)	0.014	REF	<0.001
Yes	22.7 (1,389,757)	0.025	1.02 (1.02, 1.03)		45.4 (2,779,514)	0.029	1.04 (1.03, 1.04)	

## Discussion

This large, nationally representative study provides new insight into how specific psychiatric comorbidities influence all-cause readmission risk after HF hospitalization. Schizophrenia or psychotic disorders, bipolar disorder, PTSD, and SUD were independently associated with higher 30-day and one-year readmission rates. In contrast, depression was linked to a slight reduction in 30-day readmission risk and showed no significant effect at one year, while anxiety demonstrated no significant association at either interval. These findings parallel prior nationwide research on over one million acute MI hospitalizations, which reported that schizophrenia or psychotic disorders conferred the highest 30-day readmission risk, followed by bipolar disorder, highlighting shared vulnerabilities across CV conditions [4]. Similarly, another large-scale HF study found elevated 30-day readmission risk with SUD and bipolar disorder, directly aligning with our study [17].

A key contribution of our study is the extension of analyses beyond the conventional 30-day readmission window to include one-year outcomes, offering important insight into the longer-term impact of psychiatric comorbidities on HF trajectories. While prior research has largely focused on short-term readmissions, our findings demonstrate that psychiatric conditions such as schizophrenia and bipolar disorder were associated with elevated 30-day readmission rates of 26.9% (N = 128,664) and 26.1% (N = 174,772), respectively, as well as markedly higher one-year readmission rates of 51.7% (N = 247,283) and 50.7% (N = 339,499) [4,17]. The HRs for these conditions similarly increased over time, with schizophrenia or psychotic disorders rising from HR 1.05 at 30 days to HR 1.09 at one year, and bipolar disorder from HR 1.03 to HR 1.06.

The absence of a significant association for depression at one year may reflect the complex interplay between biological mechanisms and treatment effects. Although depression is linked to autonomic dysfunction, systemic inflammation, and platelet hyperreactivity, all of which are factors that can worsen CV outcomes, its impact on long-term readmissions may be mitigated by effective management strategies [18]. For example, selective serotonin reuptake inhibitors have demonstrated benefits in reducing depressive symptoms and may contribute to improved prognosis in HF populations. This could partially explain the diminished association we observed over time. Similarly, the lack of association for anxiety at either interval may be influenced by its notably low prevalence in our dataset, potentially reflecting underdiagnosis or limitations in administrative coding. Such underrecognition may have led to misclassification and diluted any measurable relationship with readmission risk.

Our results not only highlight diagnostic differences in readmission risk but also suggest important opportunities for intervention. Prior work has shown that failure to engage in outpatient follow-up after psychiatric hospitalization significantly increases the risk of rehospitalization [19]. Those findings imply that improved discharge planning and structured follow-up could similarly help reduce readmissions in HF patients with psychiatric comorbidities. This aligns with the concept of post-hospital syndrome, which emphasizes the generalized vulnerability patients face after discharge and underscores the need for holistic, patient-centered transitional care strategies [20]. Supporting this, a national study of Medicare beneficiaries found that hospitals with higher rates of early physician follow-up after HF hospitalization had significantly lower 30-day readmission rates, reinforcing the value of timely outpatient care in improving outcomes [21].

Sociodemographic patterns in our data, which show higher readmission rates among younger, male, Medicaid-insured patients discharged to post-acute care, further illustrate how social determinants of health shape outcomes. Transitional care interventions that include early follow-up, linkage to addiction and mental health services, and sustained care coordination may help improve outcomes in these high-risk groups. Prior research emphasizes the need to match the intensity of readmission reduction efforts to patient risk rather than applying uniform strategies, as groups with psychiatric comorbidities may require tailored, resource-intensive approaches [22]. Supporting this, a meta-analysis of 18 randomized trials in older HF patients demonstrated that comprehensive discharge planning with post-discharge support can significantly reduce readmissions and improve quality of life without increasing costs, offering a model for addressing these disparities [23].

A critical gap in the current literature is the lack of clarity on the causes of readmission in the HF population. While we observed elevated all-cause readmission rates linked to certain psychiatric comorbidities, the dataset does not specify whether patients were readmitted for HF-related complications, other CV events, psychiatric crises, or unrelated conditions. Future studies must move beyond broad readmission metrics to explore specific causes of readmission across psychiatric diagnoses and guide targeted prevention and management strategies. For example, our prior analysis of CV-specific readmissions found that SUD was the only psychiatric condition associated with excess risk, while other psychiatric comorbidities were linked to reduced risk, highlighting the need for diagnosis- and cause-specific investigations to guide treatment protocols [15]. Additionally, research should also examine which diagnoses most commonly contribute to readmission within each psychiatric subgroup to identify condition-specific patterns.

Several limitations merit consideration. The NRD lacks detailed clinical data such as ejection fraction, medication adherence, or social support, all of which may influence readmission risk. Psychiatric diagnoses were identified using administrative ICD-10 codes rather than standardized clinical assessments, introducing potential misclassification and institutional variation. The low observed prevalence of anxiety may reflect underdiagnosis or coding limitations, while SUD may be more readily recognized, leading to differential representation. Additionally, the psychiatric ICD-10 code groupings have not been independently validated, and the inclusion of overlapping diagnoses introduces potential misclassification, multicollinearity, and residual confounding from unmeasured variables. We did not test interaction terms or stratify analyses by age or sex, which limits insight into subgroup-specific effects. We were unable to track patients across calendar years or capture post-discharge mortality, limiting long-term and competing risk analyses. Variation in psychiatric coding practices across participating states may have introduced geographic bias in comorbidity prevalence. While our large sample size allowed for robust detection of statistically significant differences, the observed HRs were modest, suggesting these associations may be more relevant for population-level interventions than individual-level risk prediction. Moreover, despite multivariable adjustment, the potential for residual confounding remains, and findings should be interpreted with appropriate caution given the observational design and administrative coding limitations. Finally, the dataset does not specify the causes of readmission, representing an essential priority for future research. Despite these limitations, the large sample and condition-specific focus provide valuable insights for advancing research and improving transitional care in HF patients with psychiatric comorbidities.

## Conclusions

In this large, nationally representative study of HF hospitalizations among adults, we found that bipolar disorder, schizophrenia or psychotic disorders, PTSD, and SUD were independently associated with increased all-cause readmission risk at both 30 days and one year. These associations may reflect sustained vulnerabilities related to psychiatric illness that contribute to recurrent hospitalizations well beyond the immediate post-discharge period. In contrast, depression and anxiety were not significantly associated with readmission risk after multivariable adjustment, underscoring the need for diagnosis-specific interpretation rather than a uniform approach. Integrating tailored psychiatric and addiction services into transitional care pathways, including early follow-up and comprehensive discharge planning, may help improve outcomes in high-risk adult populations. These interventions should be selectively targeted based on diagnosis-specific risk and relevant sociodemographic factors, rather than universally applied to all patients with psychiatric comorbidities. Further research is warranted to investigate the causes of readmission across psychiatric subgroups and to validate these associations in prospective adult cohorts.
